# Rapid whole-brain venous cerebral blood volume mapping using velocity-selective venous-spin-labeling with 3D GRASE

**DOI:** 10.1016/j.neuroimage.2025.121622

**Published:** 2025-11-27

**Authors:** Youngho Heo, Sung Suk Oh, Won Beom Jung, Felix W. Wehrli, Hyunyeol Lee

**Affiliations:** a Emotion, Cognition & Behavior Research Group, Korea Brain Research Institute, Daegu, South Korea; b School of Electronic and Electrical Engineering & IEDT, Kyungpook National University, Daegu, South Korea; c Medical Device Development Center, Daegu–Gyeongbuk Medical Innovation Foundation (K-MEDI Hub), Daegu, South Korea; d Department of Radiology, Perelman School of Medicine, University of Pennsylvania, Philadelphia, PA, United States

**Keywords:** Brain, Cerebral blood volume (CBV), GRASE, MRI, Velocity-selective spin labeling, Venous CBV

## Abstract

Venous cerebral blood volume (CBV_v_) is an important neurophysiological parameter and contributor to the BOLD signal mechanism. However, MRI-based, noninvasive methods for CBV_v_ mapping remain scarce, and existing approaches are limited by estimation errors resulting from a large-scale induced magnetic field or long scan times. To address these challenges, we have developed a rapid whole-brain 3D CBV_v_ mapping technique that combines velocity-selective venous-spin-labeling (VS-VSL) and a 3D gradient-and-spin-echo (GRASE) readout. In the GRASE configuration, variable refocusing flip angles were deployed for a long echo train and a short scan, with a segmented-linear center-out k-space trajectory to minimize signal modulations from alternation of gradient- and spin-echo sampling. Simulations were performed to optimize GRASE parameters for optimal scan efficiency with minimal loss of estimation accuracy, followed by experimental validations on the optimized protocol. The performance of the proposed method was evaluated in healthy subjects in terms of repeat reproducibility, and sensitivity to breath-hold and caffeine-intake challenges, stimuli known to alter blood volume in opposite directions. The optimized VS-VSL 3D GRASE sequence allowed whole-brain 3D CBV_v_ mapping in under one minute scan time with high test-retest reproducibility (R^2^/ICC = 0.88/0.93 in gray matter (GM) and 0.76/0.87 in white matter (WM)). Furthermore, the method captured the expected CBV_v_ changes in response to both breath-hold (+26.8 % (GM) and +31.2 % (WM)) and caffeine-intake (−16.3 % (GM) and −14.1 % (WM)), all presenting statistical significance (p<<0.01). The results suggest promise of VS-VSL 3D GRASE for future studies assessing spatially and temporally resolved CBV_v_ across the whole brain.

## 1. Introduction

Cerebral blood volume (CBV) refers to the total volume of blood within a given amount of brain tissue, and is typically expressed in milliliters of blood per 100 g of brain tissue, or alternatively as a percent volume fraction. As CBV is tightly coupled with cerebral blood flow (CBF) and cerebral perfusion pressure through autoregulatory mechanisms (Paulson et al., 1990), its evaluation provides important insight into normal brain function and pathological states (Donahue et al., 2006). Studies also suggest that compartmental CBV assessment (i.e., arterial CBV and venous CBV) offers distinct physiologic significance beyond total CBV measurement (Hua et al., 2019). The venous compartment is of particular interest as it is the principal source of blood-oxygenation-level-dependent (BOLD) contrast. Hence, venous CBV (CBV_v_) serves as a major modulator of the BOLD signal, and its quantification allows for understanding of brain oxygen metabolism and neurovascular coupling (Buxton and Frank, 1997; Ogawa et al., 1993).

Despite the importance, non-invasive methods for CBV_v_ mapping remain sparse. To date, three distinct MRI-based techniques have been proposed that are capable of quantifying baseline CBV_v_: quantitative BOLD (qBOLD) (Biondetti et al., 2023; Yablonskiy et al., 2013), hyperoxia BOLD (Blockley et al., 2013; Bulte et al., 2007), and velocity-selective venous-spin-labeling (VS-VSL) (Lee and Wehrli, 2020). The qBOLD approach aims to estimate both venous blood oxygenation (Y_v_) and CBV_v_ in resting-state by analyzing the time-course of the extravascular signal based on an analytical model under the assumption of static dephasing regime (An and Lin, 2002; Yablonskiy and Haacke, 1994). In the second class of methods, hyperoxia BOLD uses a hyperoxic gas mixture as a contrast agent. Since hyperoxia-induced reduction in deoxyhemoglobin increases the BOLD signal, CBV_v_ can be derived by comparing the signal change in tissue to that in a reference vein (Bulte et al., 2007).

The two approaches above (i.e., qBOLD and hyperoxia BOLD) have limitations that may prevent reliable CBV_v_ estimation. Both are prone to a large-scale, induced magnetic field (${\Delta \text{B}}_{0}$) because of data sampling at a relatively long echo time (TE) in a gradient-recalled-echo (GRE) acquisition manner, potentially resulting in signal distortions. The qBOLD technique is further challenged by the systematic coupling between CBV_v_ and Y_v_, leading to unstable and implausible solutions (Lee et al., 2018). In the hyperoxia BOLD method, hyperoxia-induced reductions in CBF (Bulte et al., 2007; Watson et al., 2000) and its possible effects on cerebral metabolic rates of oxygen (CMRO_2_) (Diringer et al., 2007; Lee et al., 2024; Rockswold et al., 2010; Xu et al., 2012) are confounding factors that may violate the model assumptions (i.e., both CBF and CMRO_2_ unchanged by hyperoxia).

More recently, a third class of methods based on the VS-VSL mechanism has been introduced to address some of the above issues. The technique employs a VS-VSL preparation module and a 3D turbo-spin-echo (TSE) readout (henceforth referred to as ‘VS-VSL 3D TSE’) so as to capture venous blood signals exclusively without ${\Delta \text{B}}_{0}$-related image distortions, yielding whole-brain 3D isotropic CBV_v_ maps with physiologically plausible values (Lee and Wehrli, 2020). Despite its advantages, the method’s relatively long scan time (~3.3 min) still limits its utility for applications tracking dynamic cerebrovascular responses to stimuli or involving tasks that may impose subject discomfort (e.g., breath-hold or hypercapnia).

In this work, we propose a new data acquisition strategy for high-speed 3D CBV_v_ mapping across the entire brain. To this end, we developed a pulse sequence that combines the VS-VSL preparation module with a 3D gradient-and-spin-echo (GRASE) readout (Oshio and Feinberg, 1991). By replacing the TSE block in VS-VSL 3D TSE with a GRASE configuration, this approach (hereafter termed ‘VS-VSL 3D GRASE’) enhances scan-time efficiency while retaining the intrinsic advantages of its parent method over qBOLD and hyperoxia BOLD. Imaging parameters were optimized using simulations, and were further validated in experiments. In vivo validation of the proposed method was performed by assessing its test-retest reproducibility in resting-state, and its sensitivity to two different stimuli (breath-hold and caffeine-intake) inducing CBV_v_ changes to opposite directions.

## 2. Materials and methods

### 2.1. Principle of VS-VSL-based CBV_v_ mapping

**Fig. 1.**
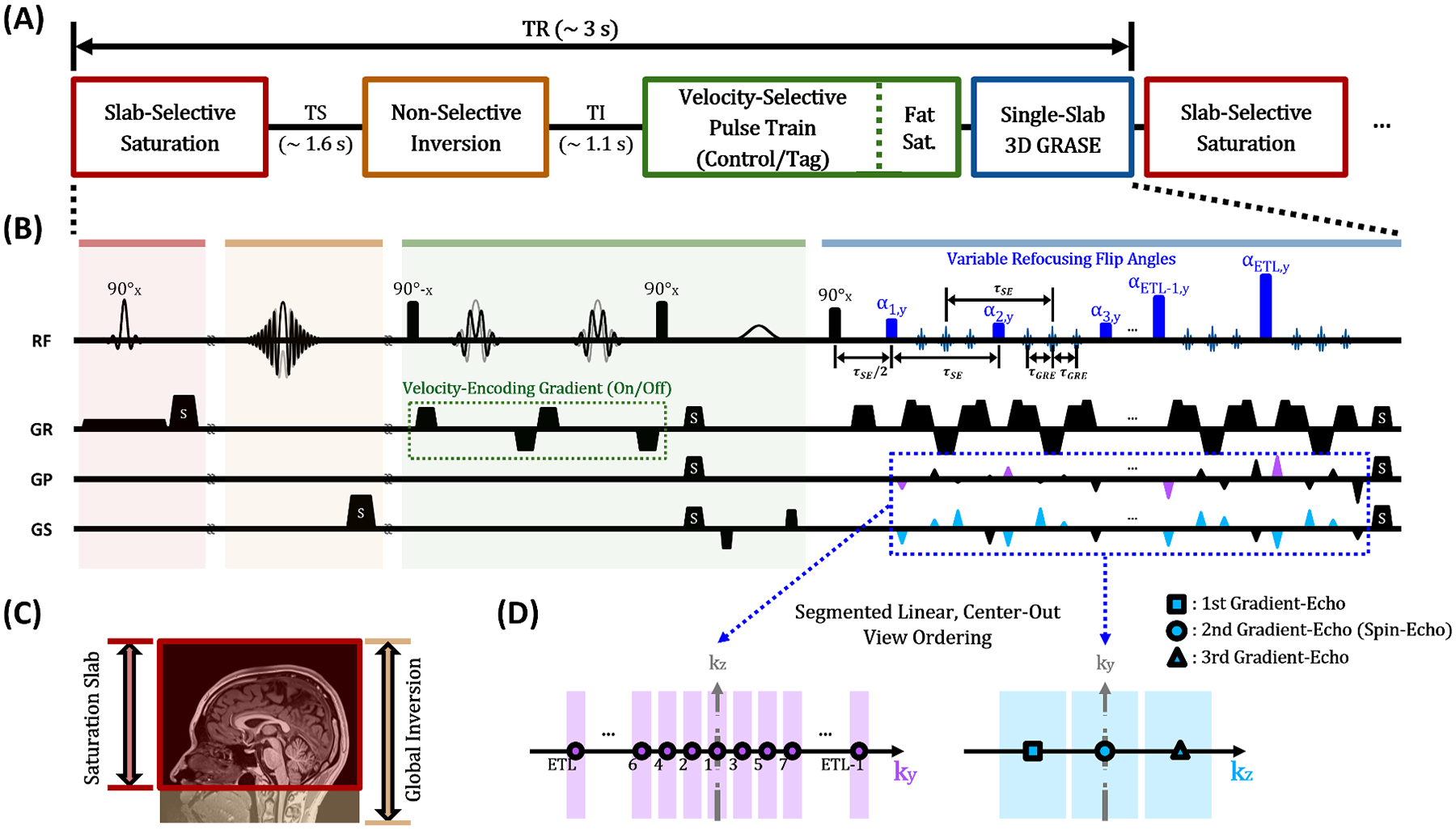
Schematic overview (A) and timing diagram (B) of the proposed VS-VSL 3D-GRASE pulse sequence, comprising four modules (indicated by colors in A). The saturation and inversion RF pulses are applied to the respective spatial positions (C), followed by the velocity-selective pulse train in either control or tag mode. Lastly, single-slab 3D GRASE with variable refocusing flip angles and segmented-linear, center-out k-space trajectory (D) is launched. Abbreviations: TS, saturation time; TI, inversion time; GR, readout gradient; GP, phase-encoding gradient; GS, slice-select gradient; S, spoiler gradient; ETL, echo train length.

The VS-VSL module is to isolate venous blood water protons from a pool of blood, cerebrospinal fluid (CSF), and brain tissue, thereby enabling CBV_v_ quantifications from signals sampled using a given readout, e.g., 3D TSE (Lee and Wehrli, 2020). Venous-spin-labeling is achieved by a composite of magnetization preparations followed by a velocity-selective (VS) pulse train (Fig. 1A, B). Specifically, sequential application of slab-selective saturation and nonselective inversion RF pulses (Fig. 1C) with the magnetization recovery times of ~1.6 s and ~1.1 s, respectively, leads to selective suppression of signals for both arterial blood and CSF, thereby isolating venous blood and brain tissue signals only. Thereafter, a VS pulse train (Norris and Schwarzbauer, 1999) that alternates between control and tag modes over each time-of-repetition (TR) is applied. Because the VS-tag mode saturates spins flowing faster than a given cut-off velocity (V_c_) while the VS-control mode acts as an all-pass filter, venous blood water protons can be selectively labeled via signal subtraction of the two VS modes. Finally, CBV_v_ maps in percent units are derived based on a simplified VS-VSL signal model, expressed as:

(1) $$CBV_{v}=\left(1-I_{tag}/I_{con}\right)\times 100(\%)$$

where $I_{\text{con}}$ and $I_{\text{tag}}$ are images reconstructed from VS-control and VS-tag signals, respectively. Details on the VS-VSL mechanism, and underlying assumptions for Eq. (1) and their validity are given in Lee and Wehrli (2020).

### 2.2. VS-VSL 3D GRASE

Fig. 1 shows a schematic and timing diagram of the proposed VS-VSL 3D GRASE technique. Following the VS-VSL preparation module described in the above subsection and a spectrally selective fat saturation RF pulse, the proposed method launches single-slab 3D GRASE. Compared with single-slab 3D TSE, the GRASE pulse sequence here presents an identical RF configuration (i.e., a spatially nonselective 90° excitation, a half echo spacing ($\tau_{\text{SE}}/2$), and spatially nonselective refocusing RF pulses with equidistant spacing ($\tau_{\text{SE}}$)), but inserts bipolar readout gradients for sampling multiple GREs with a $\tau_{\text{GRE}}$ spacing on either side of spin-echo (SE) within each two successive refocusing RF pulses, thus enabling rapid data acquisition.

The additional collection of GREs in GRASE may result in image distortions due to modulations of both amplitude and phase signals in k-space. To mitigate such issue well known to occur in conventional GRASE imaging, the present method adopted a segmented-linear, center-out view ordering (Cristobal-Huerta et al., 2018) that splits the effect of amplitude and phase modulations to orthogonal phase-encoding directions (k_y_-k_z_) in 3D k-space. Specifically, k_y_-k_z_ space is segmented into three blocks along the k_z_-direction, in which 1st and 3rd GREs are located at either periphery while 2nd GRE (i.e., SE) signals are mapped onto the center to retain relative immunity to ${\Delta \text{B}}_{0}$. Furthermore, as the GRASE pulse train evolves, echo signals are filled from the central to outer regions alternately along the k_y_-direction (Fig. 1D). This view ordering strategy yields block-wise constant phases along k_z_ and a minimal discontinuity of amplitudes along k_y_, while ensuring efficient venous blood spin labeling.

To enhance CBV_v_ imaging speed, the 3D GRASE module herein employs variable flip angles (VFAs) in the refocusing RF pulses. Compared to 3D GRASE with constant 180° refocusing flip angles, the VFA scheme leads to reduced energy deposition, and thereby permits a longer echo train and shorter scan time (Busse et al., 2008; Lee et al., 2012), while yielding a lower level of relaxation-induced signal modulations in k-space and hence less image blurring (Liang et al., 2014). In the present work, a two-step signal prescription (flat-exponential) along the GRASE pulse train was used for calculating VFAs based on an inverse solution of the Bloch equations (Park et al., 2007). Imaging acceleration was further achieved by employing an elliptical k_y_-k_z_ space with a variable-density Poisson-disc undersampling pattern (Cristobal-Huerta et al., 2019). Image reconstruction from undersampled k-space data was performed using a combined compressed sensing and parallel imaging algorithm (Daubechies et al., 2004; Lee et al., 2017; Samsonov et al., 2004).

### 2.3. Simulations

The imaging speed of VS-VSL 3D GRASE is primarily determined by the following two scan parameters: echo train length (ETL), the number of refocusing RF pulses in each GRASE pulse train, and a k-space sampling rate (η), the ratio between the sampled phase-encoding views and the total number of grids in a given k_y_-k_z_ space. Both, large ETL and low η are desirable for scan time reduction, but may result in CBV_v_ estimation errors. Specifically, with increasing ETL signal distortions are pronounced because of progressive phase incoherence among flowing spins towards the end of the GRASE pulse train, while with decreasing η image artifacts appear stronger. Hence, two separate simulations were performed to determine optimal values for ETL and η. All simulations were carried out using custom scripts in Matlab (MathWorks, Natick, MA).

#### 2.3.1. Effect of echo train length

Numerical simulations of the Bloch equation were performed based on summation of isochromats (Shkarin and Spencer, 1996). Here, two compartments were taken into account for a voxel: a static brain tissue and flowing venous blood with T_1_/T_2_ = 1300/71 ms and 1580/70 ms, respectively (Lu et al., 2004; Wansapura et al., 1999). Their relative spin densities were set to 98:2 for ground-truth CBV_v_ of 2 %. GRASE signals were calculated for both brain tissue and venous blood with the following assumptions: 1) the effect of each RF pulse on a spin isochromat is a rotation about its axis of application, 2) rectangular gradient pulses are applied in the frequency-encoding direction, 3) ${\Delta \text{B}}_{0}$ gradient is relatively much smaller than the applied encoding gradients, and thus is negligible, 4) blood spins flow along the frequency-encoding direction, and their velocity distribution follows a laminar profile with a mean velocity of 0.8 cm/s. Signal models at each time of GREs and SE along the GRASE pulse train are provided in Appendix.

VS-VSL 3D GRASE signals simulated for both VS-control and VS-tag modes were mapped onto a virtual, full elliptical k-space, respectively, using the segmented linear, center-out view ordering method described in Section 2.2 above. Finally, images for the two VS modes were obtained by Fourier transformation of the corresponding k-spaces, and subsequently subjected to Eq. (1) for CBV_v_ derivation. To evaluate the trade-off between CBV_v_ estimation accuracy and scan efficiency with respect to ETL in VS-VSL 3D GRASE, the above procedure was repeated with ETL values ranging from 20 to 84 in increments of 4. Then, both CBV_v_ estimates and scan times at each ETL were presented relative to those at ETL = 20. The simulation results were also validated via in vivo experiments (see Section 2.5.1).

#### 2.3.2. Effect of k-space sampling rate

Simulations were performed to assess the effect of k-space sampling rate (η) in VS-VSL 3D GRASE on CBV_v_ quantification accuracy, and to optimize the parameter therefrom. To this end, in vivo brain data was acquired in a full elliptical k-space using VS-VSL 3D GRASE with ETL = 54, leading to CBV_v_ maps as a reference. Scan parameters were identical to those described in Section 2.4. The fully sampled elliptical k-space (corresponding to η=75%) was retrospectively undersampled with η values of 19, 23, 30, 34, 45, and 60 %, respectively, using variable-density Poisson-disc sampling masks. For each of the subsampled k-space datasets, images were reconstructed by solving a sparse signal recovery problem. Subsequently, CBV_v_ maps obtained at each η were averaged over gray matter (GM) voxels, and compared with the reference (i.e., at η=75%). Scan time reductions relative to the reference protocol was also presented. These retrospective analyses were further validated through experiments with prospective data undersampling (see Section 2.5.1).

### 2.4. Protocols for data acquisition and processing

All experiments in this work were approved by the Institutional Review Board of Kyungpook National University, Korea. Informed written consent was obtained from each study participant. Brain scans were performed on a 3 T MRI system (Siemens Skyra, Eralngen, Germany) with a 64-channel receiver coil. The VS-VSL 3D GRASE pulse sequence was implemented in SequenceTree (Magland et al., 2016). Imaging parameters common to all experiments were: field-of-view = 220 × 220 × 180 mm^3^ (sagittal orientation), matrix size = 72 × 72 × 60, voxel size = 3 × 3 × 3 mm^3^, TR = 3 s, V_c_ = 0.8 cm/s (S/I direction) and preparation time (T_VS_) = 30 ms in the VS module, and $\tau_{\text{SE}}=5.2\;\text{ms}$ and $\tau_{\text{GRE}}=1.18\;\text{ms}$ in the GRASE readout. Additionally, high-resolution MP-RAGE images (Mugler III and Brookeman, 1990) were acquired for brain segmentation using the following scan parameters: field-of-view = 256 × 232 × 176 mm^3^ (sagittal orientation), voxel size = 1 × 1 × 1 mm^3^, TR = 2.2 s, TE = 2.45 ms, inversion time = 900 ms, flip angle = 8°, GRAPPA acceleration factor = 2 with 32 reference phase-encoding lines, and scan time = approximately 5 min.

Unless otherwise stated, all data processing, including image reconstruction, CBV_v_ mapping, and statistical comparisons, was carried out in custom MATLAB scripts. Brain segmentation on the MP-RAGE images and coregistration to VS-VSL 3D GRASE images were performed using SPM12 (Penny et al., 2011). The resliced probability maps were then thresholded at 0.6 to produce binary GM and white matter (WM) masks for quantitative analysis of obtained CBV_v_ maps. For visualization, 3D CBV_v_ maps were overlaid onto their corresponding MP-RAGE images using the MRIcron software (https://people.cas.sc.edu/rorden/mricron/index.html).

### 2.5. Experiments and analyses

Experimental studies comprised validation of simulation-determined values of ETL and η (Section 2.5.1), and validation of the optimized VS-VSL 3D GRASE-based CBV_v_ mapping protocol in terms of test-retest reproducibility (Section 2.5.2), and its sensitivity to two vascular challenges yielding opposite effects on CBV_v_: breath-holding (Section 2.5.3) and caffeine-intake (Section 2.5.4). In all statistical comparisons, a p-value < 0.05 was considered statistically significant.

#### 2.5.1. Validation of simulations

To validate the simulation findings with in vivo experiments, data were acquired in six healthy volunteers (age mean ± standard deviation (SD) = 27 ± 1 years; 4 males) using VS-VSL 3D GRASE with three different combinations of ETL and η:1) ETL = 20 and η=75% (scan time = 342 s), 2) ETL = 54 and η=75% (127 s), and 3) ETL = 54 and η=33% (57 s). Whole-brain 3D maps of CBV_v_ obtained in the three cases are presented in three orthogonal planes. For the three protocols, CBV_v_ in GM and WM voxels were averaged, respectively, in all study participants and tabulated. Furthermore, relative group-averages of GM CBV_v_ in protocols 2 and 3 were computed using protocols 1 and 2 as references, and the results were compared with those obtained from simulations described in Section 2.3.1 and Section 2.3.2, respectively. Based on the simulations and experimental validations (see Section 3.1), ETL = 54 and η=33% were chosen for all subsequent experiments.

#### 2.5.2. Repeat reproducibility

Test-retest reproducibility of the proposed, VS-VSL 3D GRASE-based CBV_v_ mapping method was assessed from datasets collected twice in 10 healthy subjects (age = 28 ± 5 years; 9 males). Here, the optimized VS-VSL 3D GRASE pulse sequence (i.e., protocol 3 in the above section) was applied. The experimental protocol consisted of a baseline scan (57 s), approximately 30-minute break outside the scanner room, and a repeat scan (57 s). CBV_v_ measured at each scan was averaged across GM and WM regions, respectively, in each subject, and their changes across the two scans were presented in box plots. Furthermore, the inter-scan agreement of CBV_v_ in GM and WM was evaluated by means of a two-tailed, paired t-test, Bland-Altman analysis, intra-class correlation coefficient (ICC), and coefficient of determination (R^2^).

#### 2.5.3. Sensitivity to vasodilatory stimulus

**Fig. 2.**
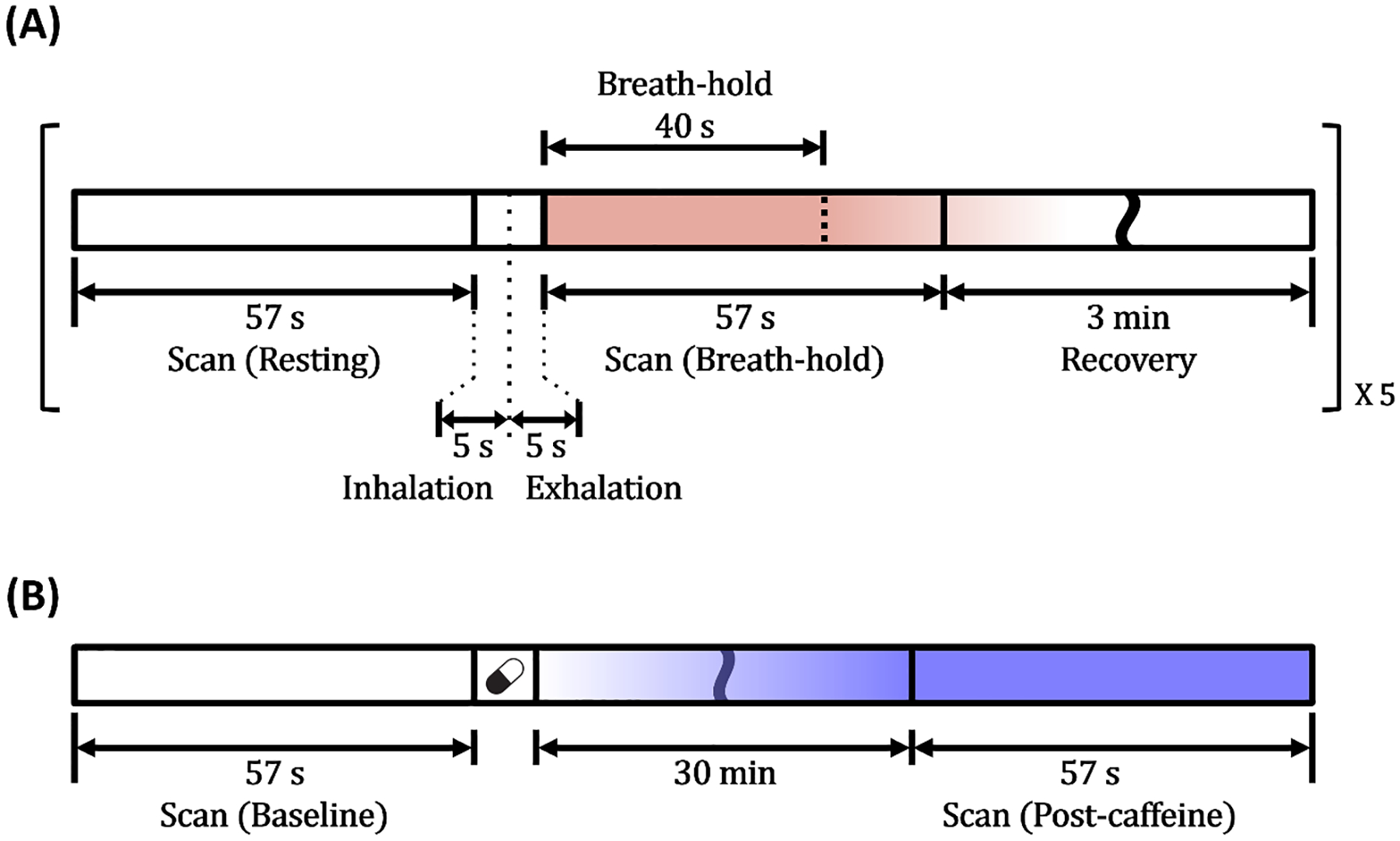
Experimental paradigms for breath-hold (A) and caffeine-intake (B) challenges for validations of VS-VSL 3D GRASE-based CBV_v_ mapping with a 57 s scan time.

Sensitivity of the new CBV_v_ mapping method to breath-holding, a potent vasodilatory stimulus, was evaluated in 10 healthy volunteers (age = 27 ± 6 years; 8 males). It is noted that while CBV changes during breath-hold have been reported with limited spatial coverage (Hua et al., 2010; Lu et al., 2003), measuring CBV_v_ responses to this stimulus across the entire brain remains unexplored. Fig. 2A illustrates the experimental paradigm, which consists of five successive repeats of the following protocol: 1) a baseline scan (57 s) during normal breathing, 2) 5 s inhalation and 5 s exhalation, 3) a scan (57 s) under 40 s suspended respiration starting at its onset, and 4) 3 min recovery. Each subject was guided under visual and auditory cues. CBV_v_ maps in a representative subject at both baseline and breath-hold states were reformatted into sagittal, coronal, and axial planes, respectively, for visual comparison. Thereafter, CBV_v_ in GM and WM regions were averaged across the five repeats in all study participants, and presented as box plots for the two respiratory conditions. Statistical significance of CBV_v_ changes in response to the volitional apnea was assessed using two-tailed, paired t-tests.

#### 2.5.4. Sensitivity to vasoconstrictive stimulus

Here, we examined sensitivity of the proposed method to a stimulus that elicits a vascular response opposite to that of apnea. To this end, 13 healthy subjects (age = 28 ± 5 years; 11 males) were scanned before and after caffeine-intake, respectively, as caffeine is an easily accessible vasoconstrictor (Addicott et al., 2009; Mathew and Wilson, 1985). All study participants were instructed to refrain from caffeine consumption for at least 24 hours prior to scanning, and verbally confirmed compliance. Fig. 2B shows the challenge paradigm, where each subject was subjected to a resting-state scan (57 s), ingestion of a single, 200 mg caffeine tablet, a 30-minute absorption period, and a post-caffeine scan (57 s). Procedures in data processing and analysis were identical to those stated in Section 2.5.3 above.

## 3. Results

### 3.1. Simulations and experimental validations

**Fig. 3.**
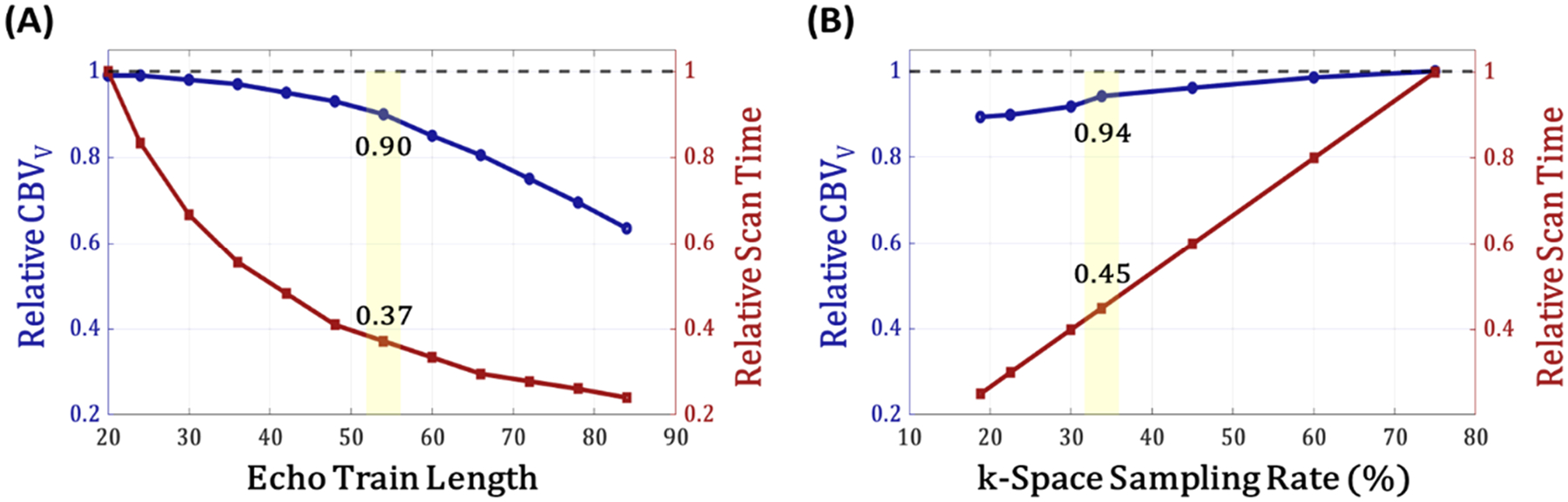
Simulations on the effects of echo train length (ETL) and k-space sampling rate (η) on CBV_v_ estimation accuracy and scan time in VS-VSL 3D GRASE. Relative CBV_v_ (blue) and relative scan time (red) are plotted against ETL (A) and η (B). CBV_v_ values and scan times corresponding to the protocols with ETL=20/η=75% and ETL=54/η=75% were used as references (dotted gray lines) in A and B, respectively. The shaded regions in both panels indicate the parameter sets (ETL = 54 and η=33%) selected for all subsequent validation experiments.

Fig. 3 shows variations of both relative CBV_v_ estimates and relative scan times as a function of ETL (Fig. 3A) and η (Fig. 3B). In Fig. 3A, a reference CBV_v_ value was obtained using ETL = 20 and η=75%, while in Fig. 3B from ETL = 54 and η=75% (corresponding to a fully sampled elliptical k-space). As ETL increases, scan times are reduced near-exponentially, but the accuracy of CBV_v_ estimation is progressively decreased (Fig. 3A). VFA-induced phase incoherence among flowing spins increases towards the later portion of the GRASE pulse train, resulting in a substantial loss of venous blood signals, and thereby, CBV_v_ tends to be underestimated with high ETL values. Similarly, decreasing η leads to a gradual loss of CBV_v_ measurement accuracy (Fig. 3B), likely due to elevated aliasing artifacts. Based on these simulations, ETL = 54 and η=33% (shaded regions in Fig. 3) were selected for VS-VSL 3D GRASE, enabling sub-minute whole-brain 3D CBV_v_ imaging.

**Fig. 4.**
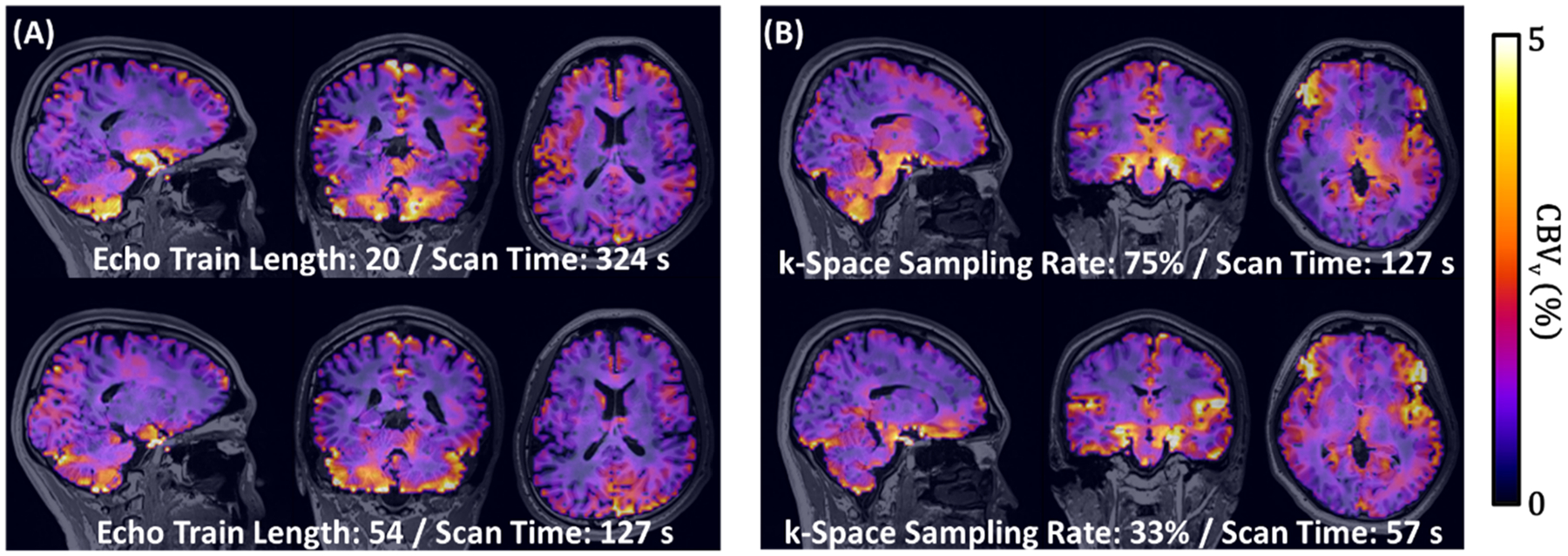
Experimental validations of the simulation-determined parameters. 3D CBV_v_ maps obtained using: (A) echo train length (ETL) of 20 versus 54, with the k-space sampling rate (η) fixed to 75 %, and (B) η=75% versus 33 % with ETL held constant to 54. Note overall consistency across all CBV_v_ images.

Fig. 4 compares 3D CBV_v_ maps obtained from VS-VSL 3D GRASE using ETL = 20 (protocol 1; scan duration = 342 s) vs. 54 (protocol 2; 127 s) with η=75% (Fig. 4A), and η=75% (protocol 2; 127 s) vs. 33 % (protocol 3; 57 s) with ETL = 54 (Fig. 4B). Supplementary Fig. S1 shows I_con_, I_tag_, and their difference images, along with the resulting CBV_v_ maps without MP-RAGE overlays, corresponding to each of the four panels in Fig. 4. While images compared are overall in good agreement, all consistently exhibit the expected CBV_v_ contrast between GM and WM regions. Table 1 summarizes CBV_v_ values in GM/WM regions measured in six study subjects across the three protocols, yielding group-averages of 2.5 ± 0.3/1.2 ± 0.2 % (protocol 1), 2.2 ± 0.3/1.3 ± 0.2 % (protocol 2), and 2.1 ± 0.4/1.2 ± 0.3 % (protocol 3). The ratios of the group-averaged CBV_v_ between protocols 1 and 2 (0.88) and between protocols 2 and 3 (0.95) are consistent with the corresponding simulation results (0.90 and 0.94; Fig. 3).

### 3.2. Repeat reproducibility

**Fig. 5.**
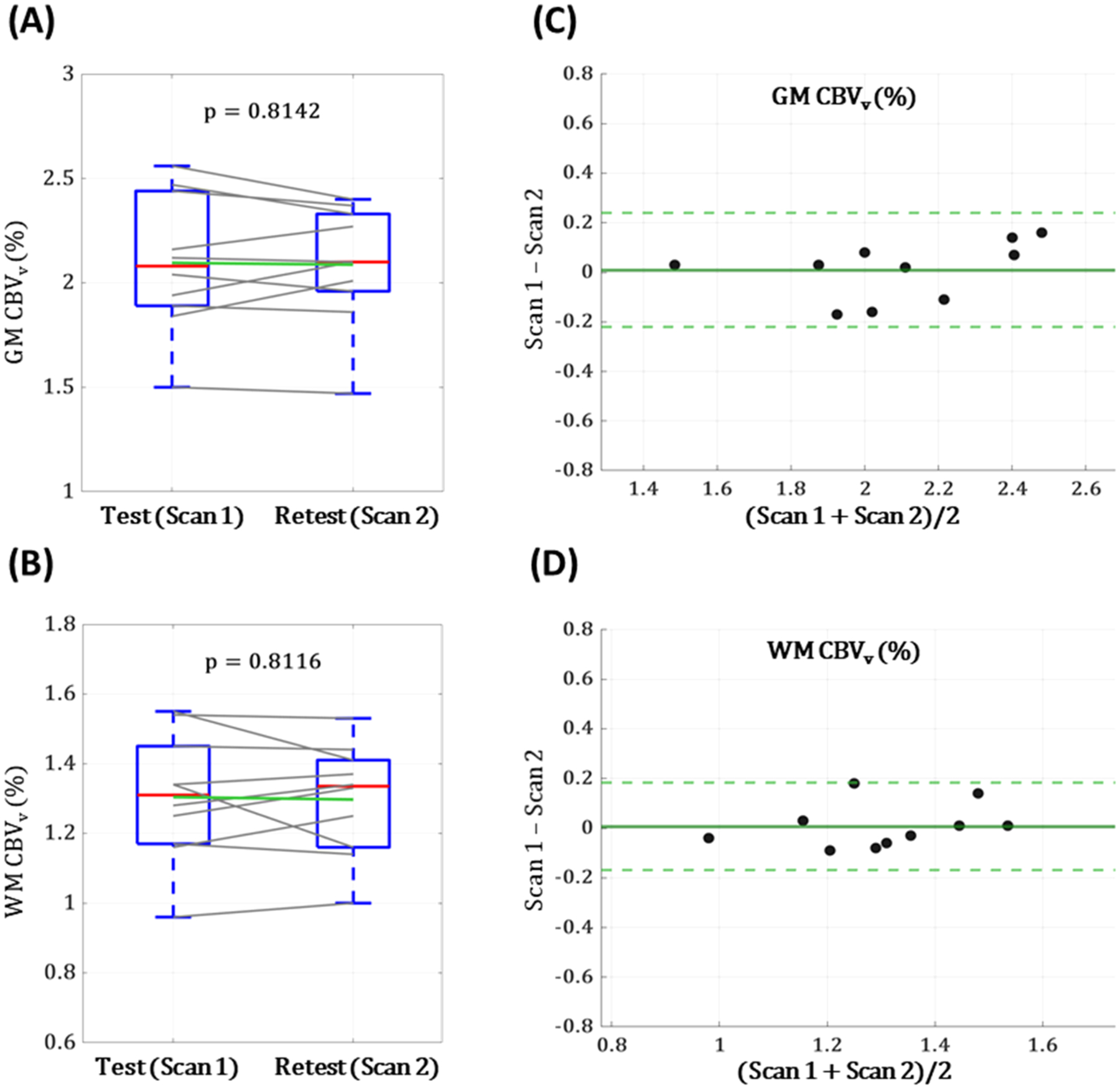
Analyses on test–retest reproducibility of CBV_v_ measurements in gray matter (GM) and white matter (WM), obtained using the optimized VS-VSL 3D GRASE pulse sequence (ETL = 54, η=33%, and scan time = 57 s). Box plots display subject-wise paired CBV_v_ values in GM (A) and WM (B) over the two scans. Red and green lines indicate group medians and means. The horizontal edges are the 25th and 75th percentiles, while whiskers outside a box are maximum and minimum of each group. In Bland–Altman plots comparing inter-scan measurements in GM (C) and WM (D), note near-zero mean bias (solid green lines), with all sample points (individual subject’s CBV_v_) falling within the 95 % limits of agreement (dashed green lines).

Box plots of regional averages of CBV_v_ across test-retest scans in ten study subjects are shown in Fig. 5A (GM) and Fig. 5B (WM). Corresponding group-averages in GM/WM were 2.10 ± 0.33/1.30 ± 0.18 % (scan 1) and 2.09 ± 0.28/1.30 ± 0.16 % (scan 2), yielding no statistical differences over the two scans (p=0.814(GM) and p=0.812(WM)). Furthermore, inter-scan R^2^ and ICC were: 0.88 and 0.93 in GM, and 0.76 and 0.87 in WM, suggesting strong agreement between the two CBV_v_ measurements. Fig. 5C and 5D display Bland-Altman plots for additional test-retest analysis, yielding a near-zero mean bias in both GM (Fig. 5C) and WM (Fig. 5D) with all data points falling within the 95 % limits of agreement. Collectively, these results indicate that the proposed method provides repeatable CBV_v_ measurements, establishing a stable baseline for detecting changes in response to physiologic challenges.

### 3.3. Sensitivity to vascular challenges

**Fig. 6.**
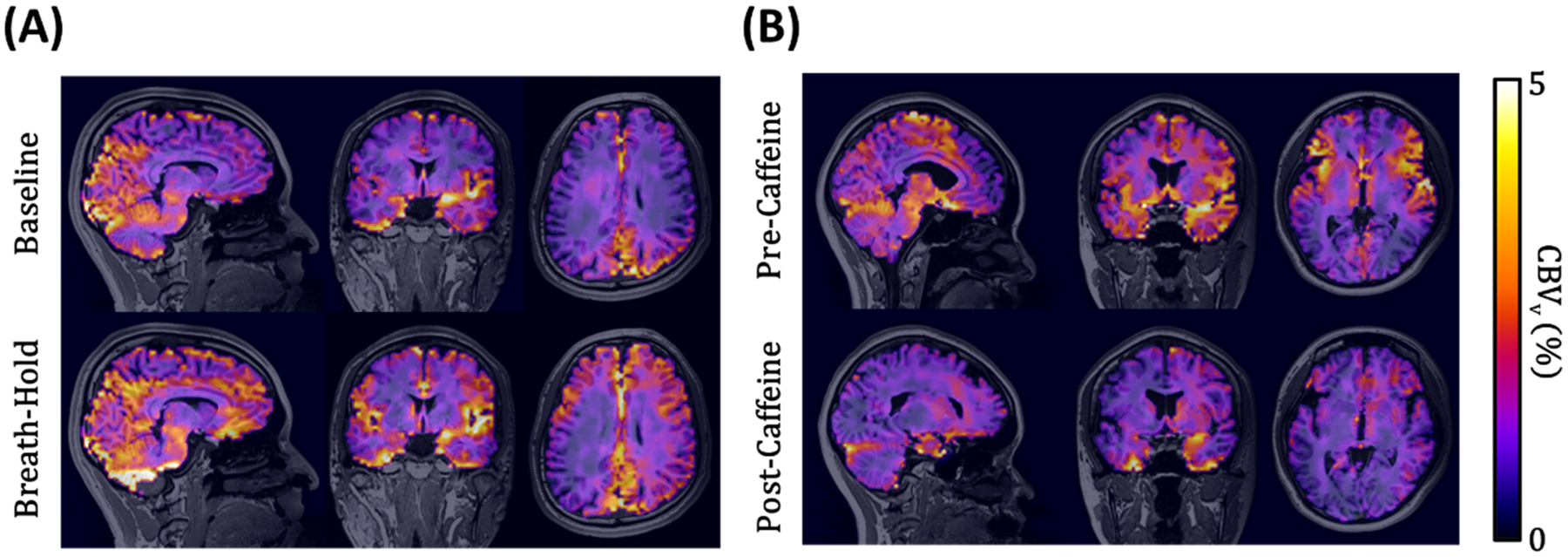
Comparison of whole-brain 3D CBV_v_ maps at baseline versus breath-hold states (A) and under pre- versus post-caffeine conditions (B), obtained using VS-VSL 3D GRASE. Note that the expected CBV_v_ changes, i.e., vasodilation (breath-hold) and vasoconstriction (caffeine), are well depicted across the entire brain.

**Fig. 7.**
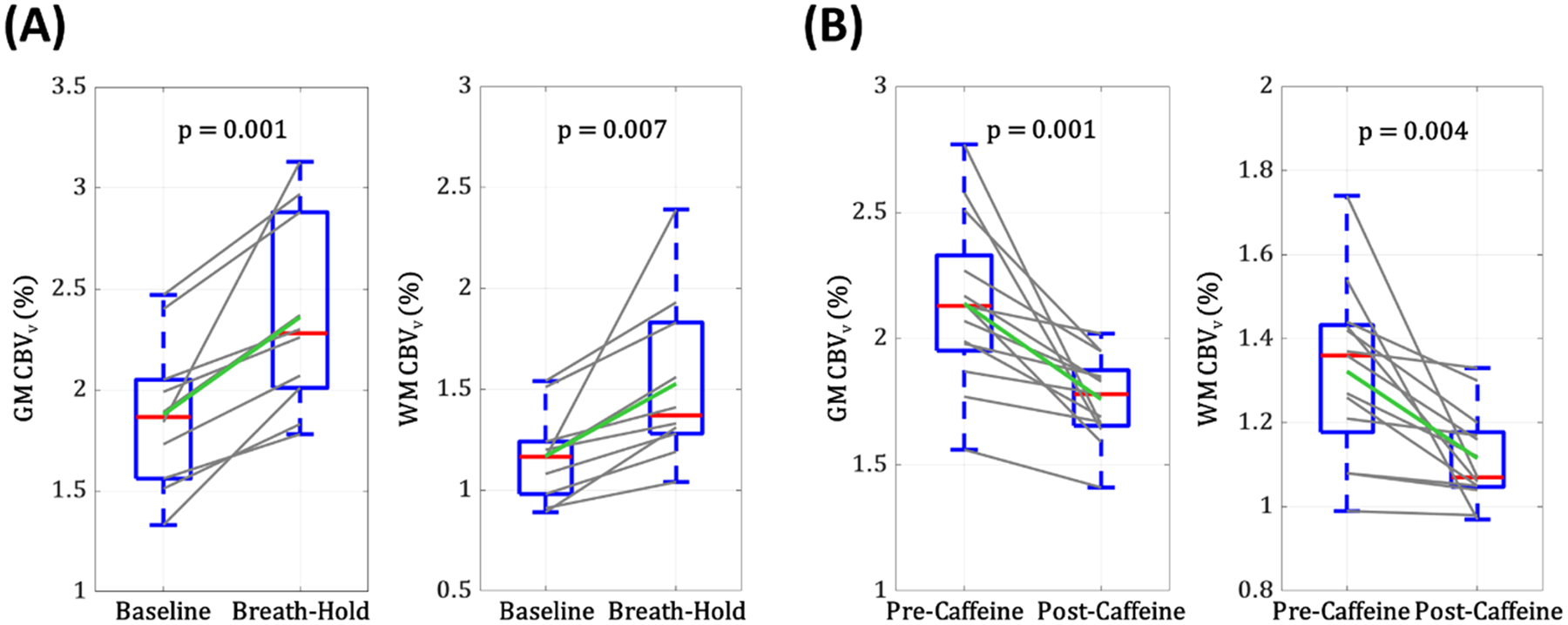
Box plots of CBV_v_ derived from the present VS-VSL 3D GRASE method in 10 subjects at baseline and breath-hold (A) and in 13 subjects under pre-caffeine and post-caffeine conditions (B). Note all study participants presented the opposing directional CBV_v_ responses to the two stimuli, leading to statistical significance of the changes. The interpretations of the box plots are identical to those stated in the caption of Fig. 5.

Fig. 6 displays CBV_v_ maps reformatted into the three orthogonal planes, obtained using VS-VSL 3D GRASE in a representative subject at rest vs. apneic states (Fig. 6A), and in another subject under pre- vs. post-caffeine conditions (Fig. 6B). Compared to baseline (i.e., resting or pre-caffeine), the expected, opposite directional CBV_v_ changes, i.e., vasodilation by breath-hold and vasoconstriction by caffeine-intake, are well depicted across the entire brain. Boxplots in Fig. 7 indicate that these vascular responses to the two challenges were consistent over all study participants. Group-mean CBV_v_ changes relative to corresponding baseline values in GM/WM were 26.8 ± 18.8 %/31.2 ± 28.4 % at apnea (n=10) and −16.3 ± 11.7 %/–14.1 ± 12.7 % at post-caffeine state (n=3), respectively. All changes were statistically significant (p<<0.01).

## 4. Discussion

In this work, we developed and validated a new MRI method, VS-VSL 3D GRASE, for rapid and noninvasive measurements of CBV_v_ across the whole brain. High-speed venous blood signal encoding was achieved by integrating VFA-based 3D GRASE into the VS-VSL contrast mechanism (Fig. 1), and further by optimizing both ETL and k-space subsampling rate in the GRASE module via simulations (Fig. 3) and experimental validations (Fig. 4). The optimized 3D GRASE allowed a sub-minute scan for full-brain CBV_v_ quantifications, enabling validation studies, in particular involving breath-hold, a challenge that would not be achievable with the prior VS-VSL-based technique (Lee and Wehrli, 2020). Performance evaluation of the new method via test-retest and two vascular challenges yielded high reproducibility (Fig. 5), and conformed to the expected bidirectional CBV_v_ changes, both qualitatively (Fig. 6) and quantitatively (Fig. 7), suggesting its potential utility in studies assessing dynamic cerebrovascular responses to a broader range of stimulation.

In addition to imaging efficiency, CBV_v_ mapping with VS-VSL 3D GRASE benefits also from relative ${\Delta \text{B}}_{0}$ immunity, a property inherited from its TSE-based predecessor (Lee and Wehrli, 2020). The GRASE readout in this work employed a minimal GRASE factor of three (i.e., two GREs and one SE within each two neighboring refocusing RF pulses) in order to retain this advantage, and ${\Delta \text{B}}_{0}$-induced signal distortions are nearly unnoticeable in the resultant CBV_v_ images (Figs. 4 and 6). One could expect higher scan efficiency with a larger GRASE factor. However, such setting results in elevated inter-echo signal incoherence and increased ${\Delta \text{B}}_{0}$ sensitivity, which potentially manifest as image artifacts and CBV_v_ quantification errors. Additionally, as the GRASE pulse train is elongated accordingly with increasing the GRASE factor, ETL would need to be adjusted to a smaller value to maintain CBV_v_ estimation accuracy (as implied by the simulations in Fig. 3A), which in turn results in a loss of scan efficiency. Given these considerations, future scrutiny on the effect of the GRASE factor is warranted.

All CBV_v_ images obtained in this study present the expected, higher vascular density of GM relative to WM (Figs. 4 and 6). While group-averaged values of 2.0/1.3 % (GM/WM) are physiologically plausible, their ratio are in good agreement with those measured using hyperoxia BOLD (2.2/1.3 % for GM/WM) (Blockley et al., 2013) and qBOLD (2.8/1.8 %) (Lee and Wehrli, 2022). The statistical analyses on test-retest datasets also indicate excellent inter-scan agreement (ICC ~ 0.9 and a mean bias ~ 0; Fig. 5), suggesting that CBV_v_ quantification from the presented method is highly reproducible.

The VS-VSL 3D GRASE-based CBV_v_ mapping protocol detected the expected vascular responses to both apnea and caffeine-intake in all study subjects (Fig. 7), with average CBV_v_ changes of 26.8 % (GM) and 31.2 % (WM). Kastrup et al. reported a 63 % increase in CBF during a 30-second breath-hold period (Kastrup et al., 1999), which corresponds to a ~21 % rise in CBV according to Grubb’s relation (${\text{CBV}\propto \text{CBF}}^{\alpha }$) with α=0.38 (Grubb Jr et al., 1974). The slightly greater CBV change observed in this study may be attributed to a longer breath-hold duration (40 s), which could yield a more pronounced vasoconstrictive effect than Kastrup’s 30-second protocol. Similarly, caffeine-induced CBV_v_ changes of −16.3/−14.1 % for GM/WM are in close agreement with those expected from the findings of (Perthen et al., 2008), who reported a CBF reduction of −34.5 % from a 200 mg caffeine dose, corresponding to a CBV decrease by −15 % under Grubb’s model.

One major assumption underlying the simplified expression of CBV_v_ in Eq. (1) is that T_2_ values of venous blood (T_2,v_) and brain tissues (T_2,b_) are approximately the same, which is valid at rest condition at 3 T However, under physiologic stimuli this assumption may not hold, potentially introducing systematic bias into CBV_v_ quantifications. Of note is that blood T_2_ changes with its oxygen saturation level (Thulborn et al., 1982). Compared with a resting state, breath-hold and caffeine-intake render Y_v_ (and also venous blood T_2_) increased and decreased, respectively (Jiang and Lu, 2022). Thus, CBV_v_ values at apneic and post-caffeine states reported in this work may have been overestimated and underestimated, respectively, according to the sensitivity analysis performed in the previous study (Lee and Wehrli, 2020). Future studies would be needed to evaluate the extent of such errors in VS-VSL-based CBV_v_ mapping that may result from stimulus-dependent alterations in blood T_2_.

Although the optimized VS-VSL 3D GRASE pulse sequence achieves whole-brain CBV_v_ mapping within a sub-minute scan, its utility still remains limited for applications tracking a relatively short-term response of cerebrovascular challenges, for example, in event-related functional MRI experiments. To further enhance temporal resolution of the present method for its applications to such paradigms, the following two strategies could be incorporated into the 3D GRASE module: reduced field-of-view imaging that selects a target brain region such as visual or motor cortices (Park et al., 2021), or dynamic MRI reconstruction methods that exploit spatiotemporal correlations with optimized k-t sampling patterns (Otazo et al., 2015; Trémoulhéac et al., 2014). Pursuing these directions would be worthwhile in future studies.

Arterial spin labeling (ASL) (Jaafar and Alsop, 2024) and vascular space occupancy (VASO) (Lu and van Zijl, 2012) fall into another class of noncontrast, quantitative cerebrovascular MRI techniques, measuring CBF and CBV, respectively. While ASL, VASO, and VS-VSL share similarities in pulse sequence structures, i.e., magnetization preparation using combinations of inversion, saturation, and/or velocity-selective RF pulses, they differ in the order, timing, and spatial coverage of these preparation modules for capturing a different compartment of the brain vasculature (i.e., arterial, whole, and venous blood pools for ASL, VASO, and VS-VSL, respectively). Hence, VS-VSL would provide information complementary to ASL and VASO in functional neuroimaging studies. Nevertheless, compared with both ASL and VASO, which have been widely explored and well established at both 3T and 7T (Alsop et al., 2015; Huber et al., 2014, 2023), VS-VSL remains a relatively new approach, and thus needs further technical development, optimization, and validation for functional applications. In this pursuit, comparative evaluation of the three methods in terms of sensitivity, spatial specificity, and SNR should be warranted.

## 5. Conclusion

We proposed a new CBV_v_ mapping strategy based on VS-VSL 3D GRASE. Optimization of the pulse sequence for both ETL and k-space sampling rate led to a sub-minute whole-brain 3D scan protocol for quantification of the parameter in absolute units, enabling validation experiments subjecting either breath-hold or caffeine-intake challenges to study subjects. The method yielded physiologically plausible values with high test-retest reproducibility, and was able to capture the expected, bidirectional CBV_v_ responses to vasoconstrictive (caffeine) and vasodilatory (breath-hold) stimuli. Further improvement could be achieved by evaluating the validity of the CBV_v_ quantification model. With fast dynamic MR imaging techniques incorporated, VS-VSL 3D GRASE may be a promising tool in studies on quantitative and functional imaging of brain states in health and disease.

## Supplementary Material

1

Supplementary material associated with this article can be found, in the online version, at doi:10.1016/j.neuroimage.2025.121622.

## Figures and Tables

**Table 1:** CBV_v_ (%) averaged across GM and WM regions in six study subjects, obtained using VS-VSL 3D GRASE with three different combinations of ETL and k-space sampling rate (η).

Subject #	Protocol 1 ETL = 20 / η = 75 % Scan Time = 342 s		Protocol 2 ETL = 54 / η = 75 % Scan Time = 127 s		Protocol 3 ETL = 20 / η = 33 % Scan Time = 57 s	
	GM	WM	GM	WM	GM	WM
1	2.1	0.9	1.7	1.0	1.7	1.0
2	2.2	1.0	1.9	1.1	1.7	0.9
3	2.5	1.1	2.2	1.4	2.7	1.6
4	2.7	1.1	2.6	1.5	2.0	1.4
5	2.4	1.2	2.3	1.4	2.3	1.0
6	2.9	1.5	2.4	1.6	2.0	1.4
Mean ± SD	2.5 ± 0.3	1.2 ± 0.2	2.2 ± 0.3	1.3 ± 0.2	2.1 ± 0.4	1.2 ± 0.3

## Data Availability

Data and code will be made available on request to the corresponding author.

## Appendix: Signal models for VS-VSL 3D GRASE

The following describes modeling of GRASE signals for numerical simulations of the Bloch equation stated in Section 2.3.1. For simplicity, the durations of the prephasing gradient (located between excitation and first refocusing RF pulses) and each of the three bipolar readout gradient lobes are set equal to $\tau_{\text{GRE}}/2$ and $\tau_{\text{GRE}}$, respectively, while the amplitudes of prephasing and readout gradient pulses are identical. It is also assumed that the prephasing gradient begins after $\tau_{\text{GRE}}$ from the RF excitation.

The signal intensity of SE ($S_{SE}$), produced at a mid-point between each two successive refocusing RF pulses in GRASE, is calculated by:

(A1)
$$S_{SE}(n)=\frac{1}{K}\sum_{k=1}^{K}\left\{\prod_{m=1}^{n}M(k,m)\right\}R_{x}\left(90^{\circ }\right)M_{i}$$

where n is the refocusing RF pulse index, K is the total number of isochromats, $R_{x}$ is the rotation matrix about an x-axis, and $M_{i}$ is the magnetization vector immediately before the 90° excitation RF pulse. With V_c_ in the VS pulse train set equal to the mean velocity of a laminar flow, $M_{i}$ for flowing spins is approximated to $[0,0,0{]}^{\text{T}}$ and ${\left[0,0,\text{exp}\left(-\frac{T_{VS}}{T_{2,v}}\right)\right]}^{\text{T}}$ in the VS-tag and VS-control modes, respectively, while $M_{i}$ for static spins is $[0,0,\text{exp}(-{\left.\left.\frac{T_{VS}}{T_{2,b}}\right)\right]}^{\text{T}}$ in both modes. M(k,m) is the magnetization vector of k-th isochromat at m-th SE. Accounting for phase accruals from both gradient pulses and flowing velocity, M is written by (Lee et al., 2012; Park and Kim, 2010):

(A2)
$$M(k,m)=T\left(\frac{\tau_{SE}}{2}\right)R_{z}\left(\phi_{S}(k)+\phi_{M}^{\prime }(k,m)\right)R_{y}\left(\alpha_{m}\right)R_{z}\left(\phi_{S}(k)+\phi_{M}(k,m)\right)T\left(\frac{\tau_{SE}}{2}\right)$$

Here, T denotes the operator representing ${\text{T}}_{1}/{\text{T}}_{2}$ relaxations during its argument interval, $R_{y,z}$ the rotation matrix about a given axis, $\alpha_{m}$ the m-th refocusing flip angle, $\phi_{S}$ the gradient-induced uniform phase dispersion of isochromats, i.e., $\phi_{S}(k)=2\pi (k-1)/K$, and $\phi_{M}$ and $\phi_{M}^{\prime }$ the flow-induced phase shift before and after each refocusing RF pulse, respectively, expressed as:

(A3)
$$\phi_{M}(k,m)=\left\{\begin{array}{ll} \frac{5}{8}\gamma v_{k}G_{R}\tau_{\text{GRE}}^{2}, & m=1\\ \frac{1}{2}\gamma v_{k}G_{R}\tau_{\text{GRE}}\left\{(m-1)\tau_{\text{SE}}+\frac{7}{4}\tau_{\text{GRE}}\right\}, & m\geq 2 \end{array}\right.$$

(A4)
$$\phi_{M}^{\prime }(k,m)=\frac{1}{2}\gamma v_{k}G_{R}\tau_{GRE}\left(m\tau_{SE}-\frac{7}{4}\tau_{GRE}\right),\;m\geq 1$$

where γ is the gyromagnetic ratio ($\sim 2\pi \cdot 42.58\;\text{MHz}/\text{T}),\nu_{k}$ is the velocity of k-th spin isochromat, and $G_{R}$ is the amplitude of the gradient pulses.

Because the bipolar gradients are symmetric about each time of SE, signal modulations due to relaxations and flow velocity in the first and third GREs appear in opposite ways, as follows:

(A5)
$$S_{GRE_{1}}(n)=\frac{1}{K}\sum_{k=1}^{K}\left\{T^{-1}\left(\tau_{GRE}\right)R_{z}\left(-\frac{1}{4}\gamma v_{k}G_{R}\tau_{GRE}^{2}\right)\prod_{m=1}^{n}M(k,m)\right\}R_{x}\left(90^{\circ }\right)M_{i}$$

(A6)
$$S_{GRE_{3}}(n)=\frac{1}{K}\sum_{l=1}^{K}\left\{T\left(\tau_{GRE}\right)R_{z}\left(\frac{1}{4}\gamma v_{k}G_{R}\tau_{GRE}^{2}\right)\prod_{m=1}^{n}M(k,m)\right\}R_{x}\left(90^{\circ }\right)M_{i}$$

It is noted that the flow velocities simulated in this work (~0.8 cm/s, chosen to represent post-capillary venules), together with practical values of $G_{R}\sim 10\;\text{mT}/\text{m}$ and $\tau_{\text{GRE}}\sim 1.2\;\text{ms}$, make the argument of $R_{z}$ in the above equations close to zero. Hence, Eqs. A-5 and A-6 can be reduced to the following simplified forms:

(A7)
$$S_{GRE_{1}}(n)=T^{-1}\left(\tau_{GRE}\right)S_{SE}(n)$$

(A8)
$$S_{\text{GRE}3}(n)=T\left(\tau_{\text{GRE}}\right)S_{SE}(n)$$

## References

[R1] Addicott MA, Yang LL, Peiffer AM, Burnett LR, Burdette JH, Chen MY, Hayasaka S, Kraft RA, Maldjian JA, Laurienti PJ, 2009. The effect of daily caffeine use on cerebral blood flow: how much caffeine can we tolerate? Hum. Brain Mapp 30, 3102–3114. 19219847 10.1002/hbm.20732 PMC2748160

[R2] Alsop DC, Detre JA, Golay X, Günther M, Hendrikse J, Hernandez-Garcia L, Lu H, MacIntosh BJ, Parkes LM, Smits M, van Osch MJP, Wang DJ, Wong EC, Zaharchuk G, 2015. Recommended implementation of arterial spin labeled Perfusion MRI for clinical applications: a consensus of the ISMRM Perfusion Study Group and the European Consortium for ASL in Dementia. Magn. Reson. Med 73, 102–116. 10.1002/mrm.25197. 24715426 PMC4190138

[R3] An H, Lin W, 2002. Cerebral oxygen extraction fraction and cerebral venous blood volume measurements using MRI: effects of magnetic field variation. Magn. Reson. Med. Off. J. Int. Soc. Magn. Reson. Med 47, 958–966. 10.1002/mrm.10148 11979575

[R4] Biondetti E, Cho J, Lee H, 2023. Cerebral oxygen metabolism from MRI susceptibility. Neuroimage 276, 120189. 37230206 10.1016/j.neuroimage.2023.120189 PMC10335841

[R5] Blockley NP, Griffeth VE, Germuska MA, Bulte DP, Buxton RB, 2013. An analysis of the use of hyperoxia for measuring venous cerebral blood volume: comparison of the existing method with a new analysis approach. Neuroimage 72, 33–40. 23370053 10.1016/j.neuroimage.2013.01.039 PMC4012834

[R6] Bulte DP, Chiarelli PA, Wise RG, Jezzard P, 2007. Cerebral perfusion response to hyperoxia. J. Cereb. Blood Flow Metab 27, 69–75. 16670698 10.1038/sj.jcbfm.9600319

[R7] Bulte D, Chiarelli P, Wise R, Jezzard P, 2007. Measurement of cerebral blood volume in humans using hyperoxic MRI contrast. J. Magn. Reson. Imaging Off. J. Int. Soc. Magn. Reson. Med 26, 894–899. 10.1002/jmri.21096 17896390

[R8] Busse RF, Brau ACS, Vu A, Michelich CR, Bayram E, Kijowski R, Reeder SB, Rowley HA, 2008. Effects of refocusing flip angle modulation and view ordering in 3D fast spin echo. Magn. Reson. Med 60, 640–649. 10.1002/mrm.21680. 18727082 PMC2760745

[R9] Buxton RB, Frank LR, 1997. A model for the coupling between cerebral blood flow and oxygen metabolism during neural stimulation. J. Cereb. Blood Flow Metab 17, 64–72. 8978388 10.1097/00004647-199701000-00009

[R10] Cristobal-Huerta A, Poot D, Vogel M, Krestin G, Hernandez-Tamames J, 2019. Compressed sensing 3D-GRASE for faster high-resolution MRI. Magn. Reson. Med 82, 984–999. 31045280 10.1002/mrm.27789 PMC6619236

[R11] Cristobal-Huerta A, Poot DH, Vogel MW, Krestin GP, Hernandez-Tamames JA, 2018. K-space trajectories in 3D-GRASE sequence for high resolution structural imaging. Magn. Reson. Imaging 48, 10–19. 29225108 10.1016/j.mri.2017.12.003

[R12] Daubechies I, Defrise M, De Mol C, 2004. An iterative thresholding algorithm for linear inverse problems with a sparsity constraint. Commun. Pure Appl. Math 57, 1413–1457. 10.1002/cpa.20042.

[R13] Diringer MN, Aiyagari V, Zazulia AR, Videen TO, Powers WJ, 2007. Effect of hyperoxia on cerebral metabolic rate for oxygen measured using positron emission tomography in patients with acute severe head injury. J. Neurosurg 106, 526–529. 17432700 10.3171/jns.2007.106.4.526

[R14] Donahue MJ, Lu H, Jones CK, Edden RAE, Pekar JJ, Van Zijl PCM, 2006. Theoretical and experimental investigation of the VASO contrast mechanism. Magn. Reson. Med 56, 1261–1273. 10.1002/mrm.21072. 17075857

[R15] Grubb RL Jr, Raichle ME, Eichling JO, Ter-Pogossian MM, 1974. The effects of changes in PaCO2 cerebral blood volume, blood flow, and vascular mean transit time. Stroke 5, 630–639. 4472361 10.1161/01.str.5.5.630

[R16] Hua J, Liu P, Kim T, Donahue M, Rane S, Chen JJ, Qin Q, Kim S-G, 2019. MRI techniques to measure arterial and venous cerebral blood volume. NeuroImage Physiol. Quantit. MRI 187, 17–31. 10.1016/j.neuroimage.2018.02.027. PMC6095829 29458187

[R17] Hua J, Stevens R, Donahue MJ, Huang AJ, Pekar JJ, van Zijl PC, 2010. Cerebral Blood Volume Changes in Arterial and Post-Arterial Compartments and Their Relationship With Cerebral Blood Flow Alteration During Brief Breath-Holding and Visual Stimulation in Human Brain. Intl. Soc. Magn. Reson. Med, p. 1127.

[R18] Huber L, Ivanov D, Krieger SN, Streicher MN, Mildner T, Poser BA, Möller HE, Turner R, 2014. Slab-selective, BOLD-corrected VASO at 7 Tesla provides measures of cerebral blood volume reactivity with high signal-to-noise ratio. Magn. Reson. Med 72, 137–148. 10.1002/mrm.24916. 23963641

[R19] Huber L, (Renzo) Kronbichler L, Stirnberg R, Ehses P, Stöcker T, Fernández-Cabello S, Poser BA, Kronbichler M, 2023. Evaluating the capabilities and challenges of layer-fMRI VASO at 3T. Apert. Neuro 3. 10.52294/001c.85117. PMC11845223 39991189

[R20] Jaafar N, Alsop DC, 2024. Arterial spin labeling: key concepts and progress towards use as a clinical tool. Magn. Reson. Med. Sci 23, 352–366. 10.2463/mrms.rev.2024-0013. 38880616 PMC11234948

[R21] Jiang D, Lu H, 2022. Cerebral oxygen extraction fraction MRI: techniques and applications. Magn. Reson. Med 88, 575–600. 10.1002/mrm.29272. 35510696 PMC9233013

[R22] Kastrup A, Li T-Q, Glover GH, Moseley ME, 1999. Cerebral blood flow–related SignalChanges during breath-holding. Am. J. Neuroradiol 20, 1233–1238. 10472977 PMC7055989

[R23] Lee H, Englund EK, Wehrli FW, 2018. Interleaved quantitative BOLD: combining extravascular R2ʹ-and intravascular R2-measurements for estimation of deoxygenated blood volume and hemoglobin oxygen saturation. Neuroimage 174, 420–431. 29580967 10.1016/j.neuroimage.2018.03.043 PMC5949279

[R24] Lee H, Kim EY, Sohn C, Park J, 2017. Rapid whole-brain gray matter imaging using single-slab three-dimensional dual-echo fast spin echo: a feasibility study. Magn. Reson. Med 78, 1691–1699. 10.1002/mrm.26910. 28921660

[R25] Lee H, Kim E-Y, Yang K-S, Park J, 2012. Susceptibility-resistant variable-flip-angle turbo spin echo imaging for reliable estimation of cortical thickness: a feasibility study. Neuroimage 59, 377–388. 21840400 10.1016/j.neuroimage.2011.07.070

[R26] Lee H, Wehrli FW, 2020. Venous cerebral blood volume mapping in the whole brain using venous-spin-labeled 3D turbo spin echo. Magn. Reson. Med 84, 1991–2003. 32243708 10.1002/mrm.28262

[R27] Lee H, Wehrli FW, 2022. Whole-brain 3D mapping of oxygen metabolism using constrained quantitative BOLD. Neuroimage 250, 118952. 35093519 10.1016/j.neuroimage.2022.118952 PMC9007034

[R28] Lee H, Xu J, Fernandez-Seara MA, Wehrli FW, 2024. Validation of a new 3D quantitative BOLD based cerebral oxygen extraction mapping. J. Cereb. Blood Flow Metab 44, 1184–1198. 38289876 10.1177/0271678X231220332 PMC11179617

[R29] Liang X, Connelly A, Tournier J-D, Calamante F, 2014. A variable flip angle-based method for reducing blurring in 3D GRASE ASL. Phys. Med. Biol 59, 5559. 10.1088/0031-9155/59/18/5559. 25170985

[R30] Lu H, Clingman C, Golay X, Van Zijl PC, 2004. Determining the longitudinal relaxation time (T1) of blood at 3.0 tesla. Magn. Reson. Med. Off. J. Int. Soc. Magn. Reson. Med 52, 679–682. 10.1002/mrm.20178 15334591

[R31] Lu H, Golay X, Pekar JJ, Van Zijl PC, 2003. Functional magnetic resonance imaging based on changes in vascular space occupancy. Magn. Reson. Med. Off. J. Int. Soc. Magn. Reson. Med 50, 263–274. 10.1002/mrm.10519 12876702

[R32] Lu H, van Zijl P, 2012. A review of the development of Vascular-space-occupancy (VASO) fMRI. Neuroimage 62, 736–742. 10.1016/j.neuroimage.2012.01.013. 22245650 PMC3328630

[R33] Magland JF, Li C, Langham MC, Wehrli FW, 2016. Pulse sequence programming in a dynamic visual environment: sequenceTree. Magn. Reson. Med 75, 257–265. 25754837 10.1002/mrm.25640 PMC4561593

[R34] Mathew R, Wilson W, 1985. Caffeine induced changes in cerebral circulation. Stroke 16, 814–817. 3901422 10.1161/01.str.16.5.814

[R35] Mugler III JP, Brookeman JR, 1990. Three-dimensional magnetization-prepared rapid gradient-echo imaging (3D MP RAGE). Magn. Reson. Med 15, 152–157. 2374495 10.1002/mrm.1910150117

[R36] Norris DG, Schwarzbauer C, 1999. Velocity selective radiofrequency pulse trains. J. Magn. Reson 137, 231–236. 10053152 10.1006/jmre.1998.1690

[R37] Ogawa S, Menon R, Tank DW, Kim S, Merkle H, Ellermann J, Ugurbil K, 1993. Functional brain mapping by blood oxygenation level-dependent contrast magnetic resonance imaging. A comparison of signal characteristics with a biophysical model. Biophys. J 64, 803–812. 8386018 10.1016/S0006-3495(93)81441-3 PMC1262394

[R38] Oshio K, Feinberg DA, 1991. GRASE (gradient-and spin-echo) imaging: a novel fast MRI technique. Magn. Reson. Med 20, 344–349. 1775061 10.1002/mrm.1910200219

[R39] Otazo R, Candes E, Sodickson DK, 2015. Low-rank plus sparse matrix decomposition for accelerated dynamic MRI with separation of background and dynamic components. Magn. Reson. Med 73, 1125–1136. 24760724 10.1002/mrm.25240 PMC4207853

[R40] Park J, Kim EY, 2010. Contrast-enhanced, three-dimensional, whole-brain, black-blood imaging: application to small brain metastases. Magn. Reson. Med 63, 553–561. 10.1002/mrm.22261. 20187162

[R41] Park J, Mugler JP, Horger W, Kiefer B, 2007. Optimized *T*_1_ -weighted contrast for single-slab 3D turbo spin-echo imaging with long echo trains: application to whole-brain imaging. Magn. Reson. Med 58, 982–992. 10.1002/mrm.21386. 17969106

[R42] Park S, Torrisi S, Townsend JD, Beckett A, Feinberg DA, 2021. Highly accelerated submillimeter resolution 3D GRASE with controlled blurring in-weighted functional MRI at 7 tesla: a feasibility study. Magn. Reson. Med 85, 2490–2506. 33231890 10.1002/mrm.28589 PMC8855518

[R43] Paulson OB, Strandgaard S, Edvinsson L, 1990. Cerebral autoregulation. Cerebrovasc. Brain Metab. Rev 2, 161–192. 2201348

[R44] Penny WD, Friston KJ, Ashburner JT, Kiebel SJ, Nichols TE, 2011. Statistical Parametric mapping: the Analysis of Functional Brain Images. Elsevier.

[R45] Perthen JE, Lansing AE, Liau J, Liu TT, Buxton RB, 2008b. Caffeine-induced uncoupling of cerebral blood flow and oxygen metabolism: a calibrated BOLD fMRI study. Neuroimage 40, 237–247. 18191583 10.1016/j.neuroimage.2007.10.049 PMC2716699

[R46] Rockswold SB, Rockswold GL, Zaun DA, Zhang X, Cerra CE, Bergman TA, Liu J, 2010. A prospective, randomized clinical trial to compare the effect of hyperbaric to normobaric hyperoxia on cerebral metabolism, intracranial pressure, and oxygen toxicity in severe traumatic brain injury. J. Neurosurg 112, 1080–1094. 19852540 10.3171/2009.7.JNS09363

[R47] Samsonov AA, Kholmovski EG, Parker DL, Johnson CR, 2004. POCSENSE: pOCS-based reconstruction for sensitivity encoded magnetic resonance imaging. Magn. Reson. Med 52, 1397–1406. 10.1002/mrm.20285. 15562485

[R48] Shkarin P, Spencer RG, 1996. Direct simulation of spin echoes by summation of isochromats. Concepts Magn. Reson 8, 253–268.

[R49] Thulborn KR, Waterton JC, Matthews PM, Radda GK, 1982. Oxygenation dependence of the transverse relaxation time of water protons in whole blood at high field. Biochim. Biophys. Acta BBA-Gen. Subj 714, 265–270. 10.1016/0304-4165(82)90333-6 6275909

[R50] Trémoulhéac B, Dikaios N, Atkinson D, Arridge SR, 2014. Dynamic MR image reconstruction–separation from undersampled (${\backslashbf k}, t $)-space via low-rank plus sparse prior. IEEE Trans. Med. Imaging 33, 1689–1701. 24802294 10.1109/TMI.2014.2321190

[R51] Wansapura JP, Holland SK, Dunn RS, Ball WS Jr, 1999. NMR relaxation times in the human brain at 3.0 tesla. J. Magn. Reson. Imaging Off. J. Int. Soc. Magn. Reson. Med 9, 531–538. 10.1002/(sici)1522-2586(199904)9:4<531::aid-jmri4>3.0.co;2-l 10232510

[R52] Watson N, Beards S, Altaf N, Kassner A, Jackson A, 2000. The effect of hyperoxia on cerebral blood flow: a study in healthy volunteers using magnetic resonance phase-contrast angiography. Eur. J. Anaesthesiol 17, 152–159. 10758463 10.1046/j.1365-2346.2000.00640.x

[R53] Xu F, Liu P, Pascual JM, Xiao G, Lu H, 2012. Effect of hypoxia and hyperoxia on cerebral blood flow, blood oxygenation, and oxidative metabolism. J. Cereb. Blood Flow Metab 32, 1909–1918. 22739621 10.1038/jcbfm.2012.93 PMC3463882

[R54] Yablonskiy DA, Haacke EM, 1994. Theory of NMR signal behavior in magnetically inhomogeneous tissues: the static dephasing regime. Magn. Reson. Med 32, 749–763. 7869897 10.1002/mrm.1910320610

[R55] Yablonskiy DA, Sukstanskii AL, He X, 2013. Blood oxygenation level-dependent (BOLD)-based techniques for the quantification of brain hemodynamic and metabolic properties–theoretical models and experimental approaches. NMR Biomed. 26, 963–986. 22927123 10.1002/nbm.2839 PMC3510357

