# Supplementary material for: Rapid whole-brain venous cerebral blood volume mapping using velocity-selective venous-spin-labeling with 3D GRASE
Source: Neuroimage. Author manuscript; Available in PMC 2026 Jan 4. (PMC12765571; doi:10.1016/j.neuroimage.2025.121622)
Supplement: 1 [file NIHMS2130939-supplement-1.docx]

**Supplementary Material**

**Manuscript ID: NIMG-25-1702**

**Title: Rapid Whole-Brain Venous Cerebral Blood Volume Mapping Using Velocity-Selective Venous-Spin-Labeling With 3D GRASE**


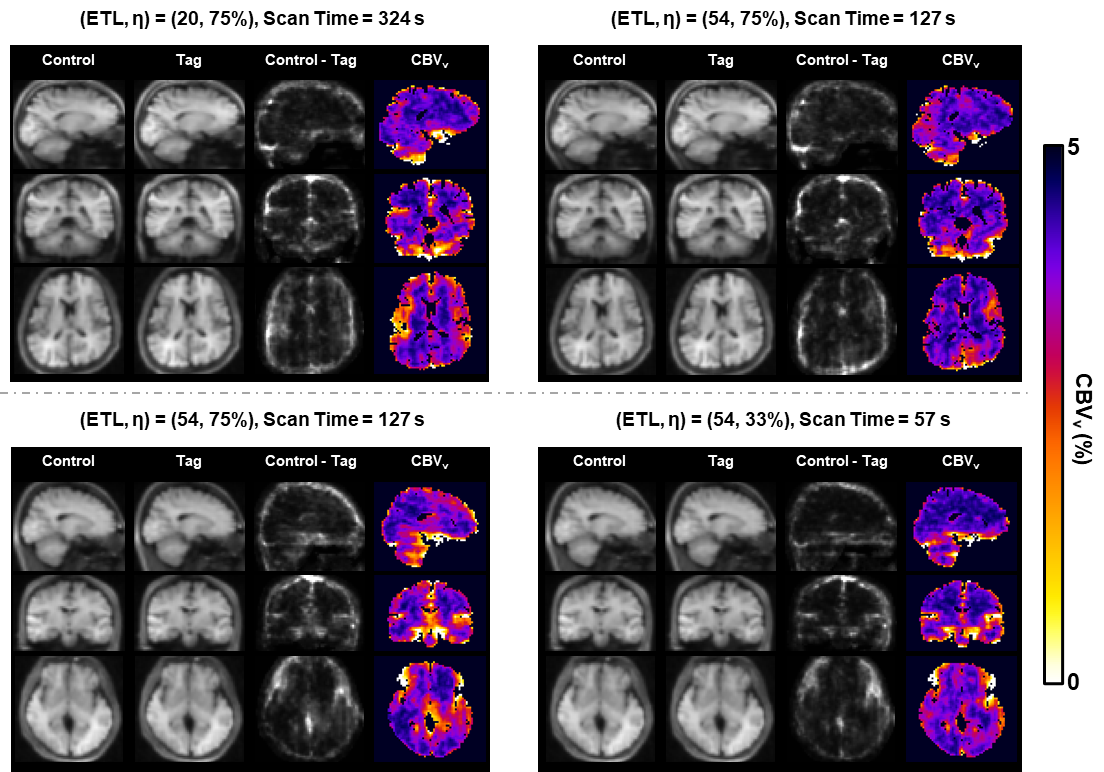


Figure S1. Whole-brain 3D images in sagittal, coronal, and axial planes, obtained from VS-VSL 3D GRASE at control and tag modes, their difference, and the resulting CBV_v_, from the first to the last columns in each panel. These panels correspond to the MP-RAGE-overlaid CBV_v_ maps in Fig. 4, acquired using different combinations of echo train length (ETL) and k-space sampling rate (η): ETL = 20 versus 54 with η fixed to 75 % (Fig. 4A), and η = 75 % versus 33 % with ETL held constant to 54 (Fig. 4B).
